# Validation of a priori candidate Alzheimer’s disease SNPs with brain amyloid-beta deposition

**DOI:** 10.1038/s41598-019-53604-5

**Published:** 2019-11-19

**Authors:** Michael Vacher, Tenielle Porter, Victor L. Villemagne, Lidija Milicic, Madeline Peretti, Christopher Fowler, Ralph Martins, Stephanie Rainey-Smith, David Ames, Colin L. Masters, Christopher C. Rowe, James D. Doecke, Simon M. Laws

**Affiliations:** 1 CSIRO Health and Biosecurity, Australian e-Health Research Centre, Floreat, 6014 Western Australia, Australia; 2 Collaborative Genomics Group, Centre of Excellence for Alzheimer’s Disease Research and Care, School of Medical and Health Sciences, Edith Cowan University, Joondalup, 6027 Western Australia, Australia; 3 Cooperative Research Centre for Mental Health, Carlton South, 3053 Victoria, Australia; 4 Department of Nuclear Medicine and Centre for PET, Austin Health, Heidelberg, 3084 Victoria, Australia; 5 The Florey Institute of Neuroscience and Mental Health, The University of Melbourne, Parkville, 3052 Victoria, Australia; 6 Department of Medicine, Austin Health, The University of Melbourne, Heidelberg, 3084 Victoria, Australia; 7 Centre of Excellence for Alzheimer’s Disease Research and Care, School of Medical and Health Sciences, Edith Cowan University, Joondalup, 6027 Western Australia, Australia; 8 National Ageing Research Institute, Parkville, 3052 Victoria, Australia; 9 School of Pharmacy and Biomedical Sciences, Faculty of Health Sciences, Curtin Health Innovation Research Institute, Curtin University, Bentley, 6102 Western Australia, Australia; 10 CSIRO Health and Biosecurity, Australian e-Health Research Centre, Herston, 4029 Queensland, Australia

**Keywords:** Genetic association study, Diagnostic markers

## Abstract

The accumulation of brain amyloid *β* (A*β*) is one of the main pathological hallmarks of Alzheimer’s disease (AD). However, the role of brain amyloid deposition in the development of AD and the genetic variants associated with this process remain unclear. In this study, we sought to identify associations between A*β* deposition and an *a priori* evidence based set of 1610 genetic markers, genotyped from 505 unrelated individuals (258 A*β*+ and 247 A*β*−) enrolled in the Australian Imaging, Biomarker & Lifestyle (AIBL) study. We found statistically significant associations for 6 markers located within intronic regions of 6 genes, including *AC103796*.*1*-*BDNF*, *PPP3R1*, *NGFR*, *KL*, *ABCA7* & *CALHM1*. Although functional studies are required to elucidate the role of these genes in the accumulation of A*β* and their potential implication in AD pathophysiology, our findings are consistent with results obtained in previous GWAS efforts.

## Introduction

Alzheimer’s disease is the most prevalent cause of dementia in elderly populations (age > 65 years). Currently affecting more than 40 million people worldwide, this number is projected to increase at least three-fold by 2050, with the continuing growth and ageing of the population. Hallmarks of disease pathology generally appear several years prior to the onset of clinical symptoms. Although the slow progression provides opportunities for preclinical therapeutic interventions, our ability to accurately detect the disease remains limited.

The accumulation of A*β* occurs at a variable rate early in the development of AD, starting over 20 years before the onset of cognitive decline and structural brain atrophy^1,2^. The process is a well-recognised histopathological hallmark of AD, and A*β* deposition is necessary for the pathologic diagnosis of the disease. However, the formation of A*β* plaques alone is not sufficient to cause cognitive dysfunction. Individuals with high A*β* accumulation but no or minimal cognitive deficits have been observed in several studies^3^. In addition, recent studies have shown that the presence of substantial A*β* deposition had low specificity for predicting the development of AD^4,5^. These observations reflect the intricate contribution of A*β* formation in the development of AD, and the need for more research in the developmental processes of the disease.

This complex pathogenesis of AD involves multiple external risk factors and comorbidities with varying susceptibilities based upon genetic backgrounds. In recent years, genome-wide association studies (GWAS) have identified more than 20 genetic risk loci robustly associated with the disease^6–10^. Large meta-analyses such as the one conducted by the International Genomics of Alzheimer’s Project (IGAP)^11^, have played a key role in enhancing our ability to predict the risk of disease onset and expanding our knowledge around the aetiology of the disease. However, most case-control GWAS have focused on identifying variants associated with the pathologic clinical diagnosis of AD compared with cognitively normal participants, even though there is a large discrepancy in the specificity in this diagnosis. Further, there is growing evidence that susceptibility to A*β*-associated decline or rate of progression may be due to either vulnerability or resilience imparted by an individual’s genetic background^12–15^.

In this study, a total of 505 patients enrolled in the Australian Imaging, Biomarker & Lifestyle (AIBL) study, were genotyped for an a priori evidence based targeted selection of Single Nucleotide Polymorphisms (SNPs), with rationale to identify possible A*β* specific variants, from a large list of AD-related candidate genes.

## Results

We conducted an association analysis using 1610 genetic markers from 505 unrelated participants of the AIBL study (258 cases and 247 controls). Comparing demographic and clinical characteristics between A*β* status, A*β*+ participants were older (72.3 [SD:6.94] vs 69.1 [SD:6.3], p < 0.0001) and were more likely to have an *APOE ε*4 allele (40% vs 12%, p < 0.0001, Table 1). As expected, there were no differences in the frequencies of males/females between A*β* groups (p > 0.05), and MCI and AD groups were more likely to be A*β*+ than A*β*− (p < 0.0001).

**Table 1:** Population characteristics.

	Total	A*β*−	A*β*+	p-value
N	505	247	258	
Male, N (%)	236	109 (46.2%)	127 (53.8%)	0.28
Female, N (%)	269	138 (51.3%)	131 (48.7%)	
Mean Age, year (SD)	70.75 (6.83)	69.12 (6.33)	72.31 (6.94)	<0.0001
*APOE ε*4 N (%)	174	41 (23.6%)	133 (76.4%)	<0.0001
Diagnosis HC (%)	374	219 (58.6%)	155 (41.4%)	<0.0001
Diagnosis MCI (%)	54	22 (40.7%)	32 (59.3%)	
Diagnosis AD (%)	77	6 (7.8%)	71 (92.2%)	
Marker PiB NAV (%)	326	157 (48.2%)	169 (51.8%)	<0.0001
Marker Flutemetamol (%)	99	42 (42.4%)	57 (57.6%)	<0.0001
Marker Florbetapir (%)	85	52 (61.2%)	33 (38.8%)	<0.0001

*P* values determined by Fisher’s test (*APOE ε*4 and Gender), t-test (age), and Chi square analyses (diagnosis). N number, HC healthy control, MCI mild cognitive impairment, AD Alzheimer’s disease, *APOE ε*4 apolipoprotein *ε*4 allele.

In preliminary analysis containing all the markers, the strongest associations (p < 1e^−8^) with amyloid status corresponded to a set of 5 SNPs (rs429358, rs769449, rs6857, rs157581, rs2075650) located within a 20 kb region containing the apolipoprotein E (*APOE*, 3.6 kb) and the translocase of outer mitochondrial membrane 40 (*TOMM40*, 12.4 kb) genes.

To enhance the identification of additional A*β* specific variants, we conducted the same association analysis including the presence/absence of the *ε*4 allele as a covariate. A total of 10 SNPs, located within 6 loci, showed nominal evidence for association (p < 5e^−3^). Results were clumped to keep only the most representative SNP per region of linkage disequilibrium, resulting in 6 independent SNPs (Table 2). Evidence of associations with previously established AD-specific loci include *AC103796*.*1*-*BDNF* (rs2049048; p = 3.62e^−04^; OR, 0.45 [95% CI, 0.29–0.7]), *PPP3R1* (rs7593613; p = 2.04e^−03^; OR, 1.59 [95% CI, 1.18–2.13]), *NGFR* (rs9908234; p = 2.45e^−03^; OR, 2.47 [95% CI, 1.38–4.43]), *KL* (rs648202; p = 2.90e^−03^; OR, 1.84 [95% CI, 1.23–2.75]), *ABCA7* (rs3764650; p = 3.07e^−03^; OR, 2.01 [95% CI, 1.27–3.2]), *CALHM1* (rs2986018; p = 4.16e^−03^; OR, 1.59 [95% CI, 1.16–2.18]), Table 2. Details of the genotyped markers and their associated p values are presented in Supplementary Table 1.

**Table 2:** Significant SNPs and associated loci.

SNP	CHR:POS	GENE	A1/A2	MAF (case/control)	OR [L95, U95]	P
rs2049048	11:27750586	AC103796.1-BDNF	T/C	0.13 (0.09/0.09)	0.45 [0.29, 0.70]	3.62e-04
rs7593613	2:68483396	PPP3R1	T/A	0.40 (0.44/0.44)	1.59 [1.18, 2.13]	2.04e-03
rs9908234	17:49499986	NGFR	G/A	0.07 (0.09/0.09)	2.47 [1.38, 4.43]	2.45e-03
rs648202	13:33635463	KL	T/C	0.13 (0.17/0.17)	1.84 [1.23, 2.75]	2.90e-03
rs3764650	19:1046520	ABCA7	G/T	0.10 (0.13/0.13)	2.01 [1.27, 3.20]	3.07e-03
rs2986018	10:105218359	CALHM1	T/C	0.24 (0.28/0.28)	1.59 [1.16, 2.18]	4.16e-03

**Figure 1:**
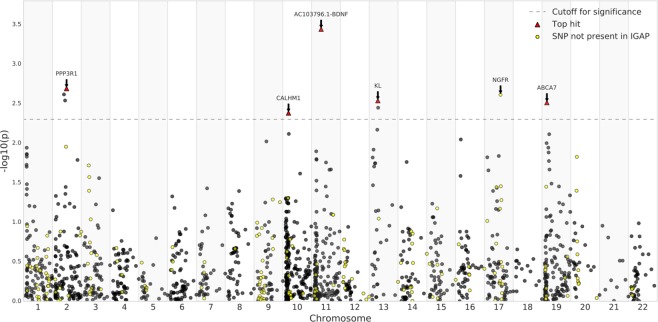
Simplified Manhattan plot for association results. The *P* values (−*log* 10) are plotted against their relative positions on each chromosome. To improve the visibility of the figure, positions correspond to the order in which the markers are located on the chromosomes. The threshold for significance was set to 5e10^−3^.

Comparing SNPs at nominal significance for A*β* in this study with the IGAP results, all but one marker (*NGFR*, rs9908234) was also found significantly associated with AD in the IGAP study. This limited overlap is explained by the use of different proxy SNPs between this study and the IGAP study. For example, the variant rs3764650 in the *ABCA7* gene was typed in this study and reached nominal significance. Although this specific variant is not present in IGAP, it is located in the same LD block (r^2^ > 0.9) as four other markers (rs73505217, rs4147911, rs4147910, rs76348507) that reached genome-wide significance in the IGAP meta-analysis. Figure 1 shows a Manhattan plot of all SNPs tested in the current study, with the -log10 of the p-value on the y-axis, chromosome on the x-axis, and dot colour representing the presence/absence of markers in the IGAP study.

**Figure 2:**
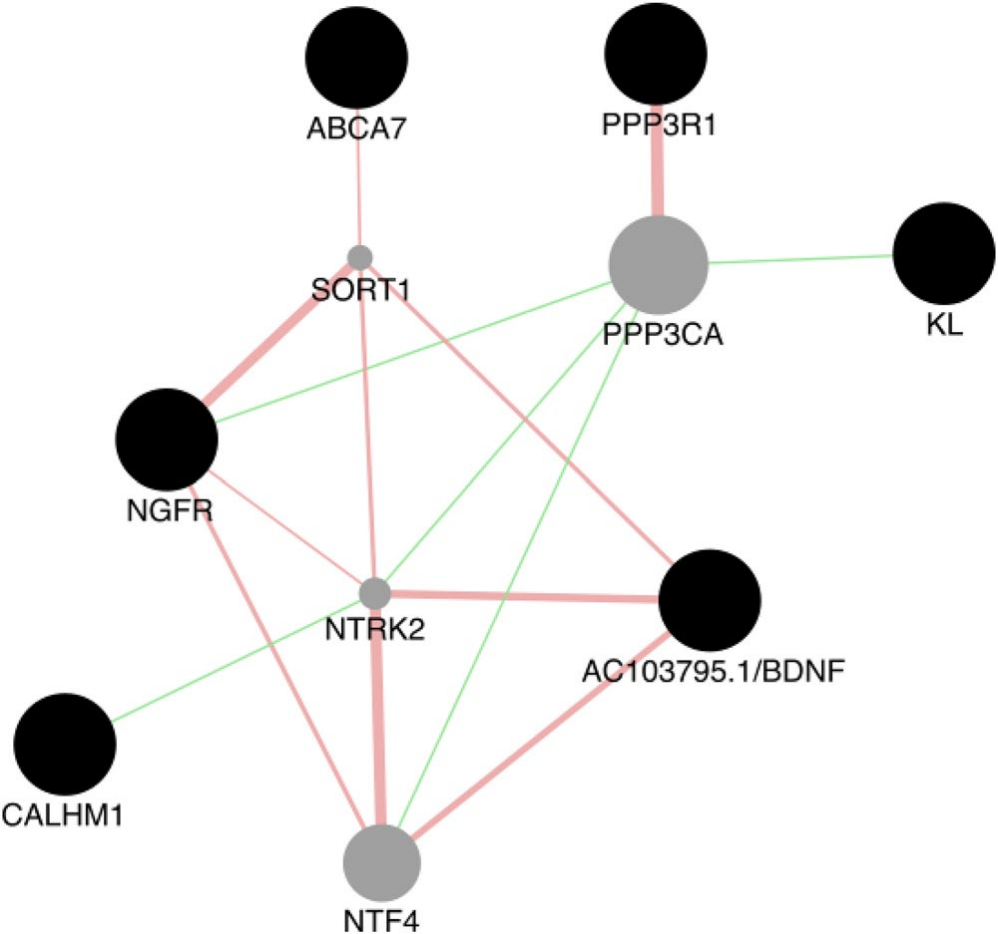
Gene-gene interaction network. The graph represents possible interactions between 6 genes in which variants associated with A*β* deposition were identified (black nodes). Indirect interactions are facilitated by the introduction of 4 external genes (gray nodes): Neurotrophin 4 (NTF4), Neurotrophic Receptor Tyrosine Kinase 2 (NTRK2), Sortilin 1 (SORT1) and Calcineurin A Alpha (PPP3CA). Edges represent the type of interactions: *physical interaction*^69–71^) are colored in red and *genetic interaction*^72^ in green. The network was generated using GeneMANIA^16^.

Lastly, we assessed the gene ontologies and functional interactions amongst the genes that reached nominal significance using the GeneMANIA resource^16^. Through the gene-gene interaction network, we were able to demonstrate the presence of physical and genetic interactions between the identified genes and several other genes with similar biological functions (Fig. 2).

## Discussion

As with most complex traits, multiple genetic variants with small and cumulative effects are likely to explain the heritability of AD. Consistent with this assumption, we identified robust associations with several previously established loci in a sample of cognitively normal and AD subjects. First, by identifying several markers located in the *TOMM40*-*APOE* region, our analysis supports the hypothesis that *APOE* on chromosome 19 is a major susceptibility gene for AD. The *APOE ε*4 allele has been associated with an increased risk of developing AD in a number of independent studies. Therefore, these results were expected but can be considered as a validation of our dataset. Aside from SNPs within the *TOMM40*-*APOE* locus, we identified a set of 6 variants at the nominal significance level showing evidence of association with A*β* status. A gene-gene interaction network revealed direct and indirect interactions amongst the 6 genes in which the genetic markers are located, suggesting a collective influence of genetic polymorphisms (Fig. 2).

The top-ranked SNP, rs7593613, was located in the regulatory subunit of the protein phosphatase B gene (*PPP3R1*), also known as calcineurin B. This marker was in high linkage disequilibrium (LD) with 2 other SNPs located on the same locus and showing significant associations with AD (rs28694054, p = 3.4e^−03^; rs11692815, p = 4.3e^−03^). Variants in *PPP3R1* have been previously reported as potential modulators of tau and phosphorylated tau levels in the presence of amyloid deposition. These changes are suspected to result in an accelerated progression of AD^17,18^. Calcineurin is involved in a number of pathways that regulate synaptic activity and neuronal excitability. Thereby, any impairment in this complex could have substantial effects and lead to pathological synaptic loss^19^.

The second top-ranked SNP identified, rs2049048, is located in the *AC103796*.*1* gene and less than 7 kb from the brain-derived neurotrophic factor (*BDNF*). Although the role of *AC103796*.*1* remains unclear, the gene overlaps *BDNF* over 20 kb and therefeore, may contibute to its function. *BDNF* is a neurotrophin involved in synaptic plasticity, neurogenesis, neuronal survival, and cognitive health^20^. Changes in BDNF levels are not specific to AD and have been reported in a number of neuropsychiatric disorders. It remains a key target for therapeutic treatment due to its pivotal role in the central nervous system^21^. Increasing evidence suggests that BDNF could modulate A*β* accumulation by decreasing A*β* formation^22^, limiting A*β*-mediated cell death^23^ and repairing A*β*-related damages^24^. Our findings indicate that a specific polymorphism in the *AC103796*.*1*-*BDNF* gene region (rs2049048; p = 3.62e^−04^) is indeed over represented in those who were A*β*+. Another variant, rs6265, has been more widely investigated and found to be associated with reduced hippocampal volume^25^ and cognitive decline^26–29^. However, contradictory results have also been reported^30,31^, suggesting a more complex relationship between *AC103796*.*1*-*BDNF* and cognition. In our analyses, rs6265, did not show a significant association with A*β* accumulation (p = 0.7).

Another notable association was found with the variant rs9908234 (p = 2.45e^−03^), located in the nerve growth factor receptor (*NGFR*) gene which encodes for a cell surface receptor for neurotrophins. A gene-gene interaction network indicated that *NGFR* has multiple indirect interaction with other genes identified in this study, including *AC103796*.*1*-*BNDF*, *ABCA7* and *CALHM1* (Fig. 2). In a recent meta-analysis of genome-wide association for migraine, rs9908234, was the most significantly associated marker with the disorder^32^. Although the link between the two conditions remains unclear, migraines are known to cause micro brain lesions^33^ which are promoting the development of MCI and AD^34,35^. In addition to the interaction of NGFR with the aforementioned genes, it also binds one of the major receptors for NGF and has also been reported to bind directly to APP^36,37^. These studies and others^38^ postulate a relationship between APP processing/A*β* accumulation and NGF/NGF receptor mediated signaling pathways that warrants further investigation. This relationship is further supported by the association of genetic variation in NGFR with A*β* accumulation in the current study.

We also report an association between the rs648202 marker and A*β* accumulation (p = 2.90e^−03^). The marker is located in the klotho (*KL*) gene, which codes for a single-pass transmembrane protein involved in cellular metabolism and has been associated with several age-related diseases. Recent studies have shown that mutations of *KL* caused systemic aging and reduced longevity in mice^34,35^. Conversely, overexpression of the gene resulted in healthier aging and prolongation of life^39,40^. Thus, as a key modulator of the aging process, klotho has become a candidate of interest for the development of novel therapeutic treatment for AD^41^. However, the role of *KL* in the development of AD remains to be defined, as a recent study showed that a functional variant in *KL*, namely *KL*-VS, had no influence on cognitive decline in preclinical AD^42^. The rs648202 variant, associated with A*β* in this study, is in linkage disequilibrium (D’ = 1.0) with the *KL*-VS variants (rs9527025/rs9536314), however they are not highly correlated (r2 = 0.03).

The SNP, rs3764650, located in an intron of the *ABCA7* gene showed a moderate association with A*β* accumulation (p = 3.07e^−03^). This specific variant was identified as one of the main susceptibility loci for late-onset AD in two independent cohorts^6,43^. Furthermore, recent studies have shown that rs3764650 was associated with cortical and hippocampal atrophy in cognitively normal and mild cognitive impairment (MCI) subjects^44^ as well as with memory decline in MCI and late-onset AD patients^45^. In addition, *ABCA7* has been identified as a major mediator of phagocytic clearance of A*β*^46^, which supports the reported association.

In *CALHM1*, the polymorphism rs2986018 showed marginal evidence for association (p = 4.16e^−03^). Several genetic epidemiological studies have suggested that rs2986017, a marker located in *CALHM1* and within the same LD block as rs2986018, could influence age at onset of AD^47–49^. Although the underlying mechanisms by which *CALHM1*, which codes for a calcium channel, modulates AD’s pathogenesis remain unclear, it has recently been identified as a repressor of A*β* accumulation, in cell lines and *in vivo*^50^. These findings indicate that *CALHM1* is potentially involved in A*β* degradation in the brain, a molecular mechanism highly relevant to AD’s pathogenesis.

In summary, although the present study was subject to a lack of power due to the limited number of cases and controls available, it provides suggestive evidence for the implication of several genes previously hypothesised to have a role in the development of AD, through the *a priori* evidence based approach employed for marker selection. Therefore, replication analyses in independent samples is warranted to confirm our findings and increase the significance of true associations. Whilst the nature of the marker selection employed in this study is a potential strength, the biased nature of this selection may have resulted in the exclusion of, as yet unknown, A*β*-associated genetic variants. As such further unbiased approaches in larger sample sizes is also warranted. Finally, the approach undertaken here used a cross-sectional analysis of a dichotomised sample. The use of a continuous variable of A*β*-burden or longitudinal rates of accumulation may identify other genetic variants of interest.

## Conclusion

With the increasing numbers of traits examined through genetic association analyses, it has become increasingly clear that individual genetic components are insufficient to explain complex phenotypes such as Alzheimer’s disease. Instead, such traits are most likely modulated by the collective influence of tens or even hundreds of genetic loci with small individual effects. In this study we identified 6 variants associated with the accumulation of A*β*, a key process in the pathogenesis of AD. The identified variants are located in the intronic regions of 6 distinct loci that are involved in major neurological and neurocognitive functions. Further studies are needed to fully understand the role of these variants in the AD’s pathogenesis. However, this study opens doors to the investigation of novel biological targets for AD treatment to be considered in future studies.

## Methods

### Participants

Data from the AIBL study, a prospective longitudinal study of ageing, is presented here. The AIBL study design, enrolment process, neuropsychological assessments and diagnostic criteria have been previously described^51^. Of the 1572 participants enrolled into the AIBL study 1416 of these underwent genetic analysis using the methodologies described below. Participants were classified as MCI^52^ or AD^53^ when the clinical criteria for diagnosis of was met. In the absence of these diagnoses a classification of cognitively normal (CN) was given by a clinical review panel, blinded to Amyloid-*β* status. Ethics approval for the AIBL study and all experimental protocols was provided by the ethics committees of Austin Health, St Vincent’s Health, Hollywood Private Hospital and Edith Cowan University. All experiments and methods were carried out in accordance with the approved guidelines and regulations and all volunteers gave written informed consent before participating in the study.

### SNP selection, genotyping and quality control

A thorough literature review was conducted in PubMed to identify genes with an a priori evidence of association with AD risk, cognitive performance, pathological characteristics (i.e. A*β*/tau, atrophy), candidate peripheral/CSF biomarkers, hypothesised pathomechanisms (e.g. A*β* clearance/metabolism) and other AD related biological pathways or comorbidities (e.g. endocytosis, cholesterol metabolism, steroidogenesis, diabetes/insulin resistance, cardiovascular disease). This resulted in the selection of an *a priori* candidate list of 270 genes. The final selection of 2088 genetic markers across these loci was based on prior phenotypic association and/or extended coverage of each loci. The list of genetic variants is available in Supplementary Table 2.

Genotype data was obtained from 1416 samples from the AIBL cohort using using a combination of an Illumina GoldenGate array containing 1536 markers and multiple TaqMan^®^ OpenArray™ assays.

The GoldenGate array was performed by the Beijing Genomics Institute (BGI, Shenzhen, China) as per manufacturer’s protocols. OpenArray™ assays were developed using inventoried or custom designed TaqMan^®^ genotyping assays, whilst TaqMan^®^ assays were used for *APOE* genotyping (rs7412, assay ID: C____904973_10; rs429358, assay ID: C___3084793_20; Life Technologies, Carlsbad, CA) using the TaqMan^®^ GTXpress^®^ Master Mix (Life Technologies). All TaqMan^®^ and OpenArray™ assays were performed on an Applied Biosystems™ QuantStudio™ 12 K Flex Real-Time PCR system using the manufacturer’s instructions.

Genotype data was prepared by removing markers with a genotyping call rate below 95% and a minor allele frequency (MAF) of at least 0.05. In addition, markers not in Hardy-Weinberg equilibrium (p < 10^−4^) were removed. Samples identified with discordant sex information and samples with a call rate below 95% were discarded. Approximately 65% of samples (N = 918) and over 77% of genetic markers assayed (N = 1610) reached QC procedures.

This study limited its analyses to a subset of 505 participants who had previously undergone positron emission tomography (PET) to assess neocortical A*β* burden. PET imaging was performed with three different A*β*-imaging radiotracers 11C-Pittsburgh Compound B (PiB), 18F-florbetapir (FLUTE) or 18F-flutemetamol (FBP). Methodology for each tracer has been previously described in^54^. Briefly, standardised uptake values (SUVs) were calculated via summing spatially normalised PET images sampled using a narrow cortical regions of interest template using CapAIBL^®^, a web-based, freely available software^55,56^. The SUVs were then scaled to each tracer’s recommended reference regions to define the SUV ratio (SUVR). Reference region for PiB was the cerebellar cortex^57,58^, for FLUTE the pons^59^ and for FBP the whole cerebellum^60^. All participants were then classified to a dichotomous A*β* deposition phenotype, being either high (A*β*+; n = 258) or low (A*β*−; n = 247), based on each tracer-specific neuropathology established thresholds (PiB: 1.4 SUVR, FLUTE: 0.62 SUVR and FBP: 1.05 SUVR)^58–60^. Despite displaying different dynamic ranges and subtle differences in the uptake and selectivity of the probes^61,62^, head to head comparisons of the three radiotracers have previously shown >98% concordance in their classification as PET A*β*+ and PET A*β*−^63–65^.

### Association analyses

To identify associations between genetic marker and the dichotomous A*β* status, we performed a logistic regression using PLINK2^66^. The analyses incorporated age, gender and the presence/absence of the *ε*4 allele as covariates. Considering the limited sample size of the data set and the targeted nature of the study, we used the following approach to identify an adequate threshold for significance. To estimate an empirical threshold for significance, we measured the distributions of the P-values of the variants and defined a threshold, *Psig*, as the 99th percentile (1-*α*) at a significance level of *α* = 0.01. We calculated *Psig* using the Harrell–Davis distribution-free quantile estimator^67^.

## Supplementary information

Supplementary Table 1

Supplementary Table 2

## Data Availability

All data and samples used in this study are derived from the Australian Imaging, Biomarkers and Lifestyle (AIBL) Study^68^. All AIBL data, and that specific to this study, is publicly accessible to all interested parties through an Expression of Interest procedure and is governed by the AIBL Data Use Agreement, for more information please see https://aibl.csiro.au/awd/.
